# Towards N–N-Doped Carbon Dots: A Combined Computational and Experimental Investigation

**DOI:** 10.3390/ma15041468

**Published:** 2022-02-16

**Authors:** Chiara Olla, Stefania Porcu, Francesco Secci, Pier Carlo Ricci, Carlo Maria Carbonaro

**Affiliations:** 1 Department of Physics, University of Cagliari, Cittadella Universitaria, I-09042 Monserrato, Italy; stefania.porcu@dsf.unica.it (S.P.); carlo.ricci@dsf.unica.it (P.C.R.); 2 Department of Chemistry and Geological Science, University of Cagliari, Cittadella Universitaria, I-09042 Monserrato, Italy; fsecci@unica.it

**Keywords:** carbon dots, nitrogen doping, Raman, photoluminescence, DFT, hydrazines

## Abstract

The introduction of N doping atoms in the carbon network of Carbon Dots is known to increase their quantum yield and broaden the emission spectrum, depending on the kind of N bonding introduced. N doping is usually achieved by exploiting amine molecules in the synthesis. In this work, we studied the possibility of introducing a N–N bonding in the carbon network by means of hydrothermal synthesis of citric acid and hydrazine molecules, including hydrated hydrazine, di-methylhydrazine and phenylhydrazine. The experimental optical features show the typical fingerprints of Carbon Dots formation, such as nanometric size, excitation dependent emission, non-single exponential decay of photoluminescence and G and D vibrational bands in the Raman spectra. To explain the reported data, we performed a detailed computational investigation of the possible products of the synthesis, comparing the simulated absorbance spectra with the experimental optical excitation pattern. The computed Raman spectra corroborate the hypothesis of the formation of pyridinone derivatives, among which the formation of small polymeric chains allowed the broad excitation spectra to be experimentally observed.

## 1. Introduction

In the research of luminescent materials free of toxic and dangerous elements such as heavy and rare earth metals, a new class of carbon nanoparticles has been widely studied for its outstanding properties, which range from high quantum yield (QY) to easy and low-cost synthetic methods [1,2,3,4]. Carbon Dots (CDs) are generically defined as quasi-spherical nanometric particles with a core-shell structure composed by a graphitic carbon core and a disordered surface domain with oxygen and nitrogen-containing functional groups and moieties [5,6]. Thanks to this peculiar structure, CDs present highly desirable properties such as water dispersibility, low toxicity and tunable and efficient photoluminescence (PL), which makes them greener counterparts of heavy metals and semiconductor quantum dots [7,8].

The origin of this last property is still an issue in question. Generally, their excitation-dependent photoluminescence is ascribed to quantum confinement of the nanometric graphitic core and/or to the presence of fluorescent molecules and surface defects, depending on the synthesis procedure (Vide Infra) [9,10,11,12,13].

Nevertheless, the mix of optical properties and high biocompatibility brought about the rise of an incredible number of works focusing on CD applications on lighting, photocatalysis, molecular and metal ions sensors, and bioimaging [14,15,16,17,18,19,20,21]. A range so vast of applications relies on synthetic conditions and choice of the right precursor in order to achieve the desired properties.

The basic requirement to synthesize CDs is the use of a compound with a carbon skeleton. Given this premise, two approaches to synthesize CDs are usually implemented: top-down and bottom-up methods. The top-down approach was adopted first, consisting of the production of CDs through chemical or physical cutting (laser ablation, arc-discharging) of bulk carbon sources (graphite, carbon soot) [22]. Obtained CDs generally present high ordered structures with more homogenous samples in size and distribution. In this case, the emission properties are expected to be related to the quantum confinement effect [9]. However, the use of specific instrumentation and the need for post-synthesis treatments to reach competitive QY makes the whole procedure expensive and disadvantageous. Thus, bottom-up synthetic routes have rapidly become dominant [23,24]. 

Bottom-up methods start from small molecule or polymeric precursors to reach an easy and low-cost treatment via combustion, pyrolysis, solvothermal or microwave assisted pyrolysis. All these thermal treatments may have a different target temperature, working pressure and synthesis time, producing carbon structures with a higher degree of disorder as compared to top-down obtained nanoparticles. In the bottom-up approach, optical properties are mostly related to the presence of surface state (functionalization) or formation of fluorescent molecules [9,25]. Among bottom-up approaches, the hydrothermal method provides an efficient and scalable route to synthesize carbon particles at milder conditions from organic acids, amines, saccharides and their derivatives [26,27]. In this case, the proposed formation mechanism of CD is related to polymerization, aromatization, nucleation and growth of precursors in the water medium [27,28,29,30,31,32,33,34]. A comparable mechanism was also reported for other bottom-up synthesis [35].

Furthermore, it is possible to easily modify CD properties via doping. Recently, N-doping was widely explored because of its exceptional results in enhancing PL and tuning PL wavelengths. N-doped CDs can contain embedded moieties, surface functional groups (amines and amides) and heteroaromatic rings (pyridinic, pyrrolic and graphitic nitrogen) [18]. Heterocyclic structures are obtained by the chemical reaction of amines and different carbonyl or carboxylic compounds such as citric acid (CA). It was reported that during the hydrothermal synthesis of CA and urea, CA self-assembled into a sheet-like structure followed by the condensation through intermolecular de-hydroxylation. During this process, nitrogen can enter in the system, generating a pyrrolic structure and forming N-doped CDs [32]. A similar reaction scheme was assumed in the hydrothermal treatment of CA and ethylenediamine (EDA) [28]. In those cases, the embedding of citrazinic acid (CZA), 5-Oxo-1,2,3,5-tetrahydroimidazo-[1,2-α]-pyridine-7-carboxylic acid (IPCA) and related macro molecules was reported as the origin of the photoluminescence properties of CDs [27].

The role of nitrogen in the optical properties was widely studied both from an experimental and computational point of view [32,36,37,38]. Nevertheless, most of these studies are focused on N-doping from the most popular amine precursors and, consequently, investigate the carbon–nitrogen bond in the form of pyrrolic, pyridinic and graphitic nitrogen inside the carbon network. The nitrogen–nitrogen bond is not generally considered, and a few papers about this possible bonding are reported in the literature concerning the use of hydrazines as a CD precursor or, due to their high reduction power, for modifying the bonding type of N-atoms in formed CDs which enhance their QY [39,40,41,42,43,44]. Indeed, the simplest organic compounds that present this kind of bond are represented by hydrazines, which are largely used in pharmaceutical and agrochemical industries [45,46].

Hydrazine structures are characterized by a single covalent N–N bond which carries from one up to four alkyl or aryl substituents. Hydrazines are highly toxic for human health, and, among their wide range of applications, CDs have been adopted as a fluorescent sensor for this class of compounds [47,48,49,50,51,52,53,54]. 

Despite its toxicity, Hydrazine represents the simplest molecule that presents an N–N bond, and we exploited this as a proof of concept for the possible development of N–N doping in CDs. 

Inspired by these previous works [39,40,41,42,43,44] and by the results on the citric acid plus amines reaction [27], we decided to study the possible formation of heterocyclic N–N-containing compounds as possible molecular seeds for the formation of CDs. For these reasons, a combined experimental and computational approach was adopted to investigate the optical features of hydrothermally produced citric acid and hydrazine-related CDs, showing that the formation of pyridinone structures could explain the excitation-dependent emission properties. As compared to a citric acid–urea CD reference sample, a larger relative contribution in the green region of the optical emission spectrum was obtained, suggesting promising application for these systems.

## 2. Materials and Methods

N-doped CDs were synthesized via a simple single-step hydrothermal route using citric acid monohydrate as a precursor (CA; purity ≥0.99, Sigma-Aldrich, St. Louis, MO, USA), which was reacted with selected hydrazines as nitrogen sources: (i) hydrazine hydrate (Hy; Sigma-Aldrich, St. Louis, MO, USA), (ii) phenylhydrazine (P-Hy; Sigma-Aldrich, St. Louis, MO, USA), (iii) N,N-dimethylhydrazine (DM-Hy; Sigma-Aldrich, St. Louis, MO, USA). For each synthesis, 0.77 g CA (3.66 mmol) were combined with the nitrogen source in a 1:1 molar ratio (Hy, 120 μL; DM-Hy, 270 μL; P-Hy, 360 μL; urea, 0.22 g). The reagents were dissolved in 20 mL of distilled water and the obtained solutions were transferred into a Teflon-lined stainless-steel autoclave (volume 30 mL) and loaded in a hot air oven at 180 °C for 6 h. It was not possible to measure the actual pressure inside the reactor, but, since the typical range of hydrothermal synthesis is 0.3–4 MPa and the steam temperature of water at 180 °C is about 1 MPa, we estimated the pressure inside the autoclave to be about 1 MPa. Autoclaves were cooled to room temperature in order to collect the resulting solutions, which were centrifugated at 4000 rpm and filtered using a 0.22 μm membrane to remove the larger aggregates from the final products. The synthesized samples are indicated, in the following, as CA-urea, CA-Hy, CA-DM-Hy and CA-P-Hy respectively.

Surface Enhanced Raman spectroscopy (SERS) measurements were obtained in back scattering geometry with a micro-Raman scattering confocal system (SOL Confotec MR750, SOL instruments Ltd., Minsk, Republic of Belarus) equipped with a Nikon Eclipse Ni microscope (Nikon Instruments Europe BV, Amsterdam, The Netherlands). The samples were excited at 532 nm (IO Match-Box series laser diode, Vilnius, Lithuania) and collected with a spectral resolution of 0.6 cm^−1^ (average acquisition time 50 s, average number of acquisitions 3, sensor temperature −23 °C, objective Olympus 100×, grating with 600 grooves/mm, power excitation 3 mW). SERS supports were ITO glasses coated with silver nanoparticles (S-Silver SERS substrates, Sersitive, Warsaw, Poland). 

UV-Vis-NIR absorbance and transmittance spectra were collected by an Agilent Cary 5000 spectrophotometer (Agilent, Santa Clara, CA, USA) with a spectral bandwidth of 2 nm in the 200–800 nm range. All the samples were diluted with distilled water with a dilution factor of 10^−4^ (%*v*/*v*) to avoid reabsorption effects and put in quartz cuvettes with a 1 cm path length. Baseline corrections were performed on all spectra. 

Three-dimensional fluorescence mapping of samples was performed using a spectrofluorometer, the Fluoromax-4 by Horiba Jobin Yvon (Kyoto, Japan), with a 150 W Xenon lamp as the excitation source. The maps were collected with an excitation range of 250–500 nm and an emission range of 350–600 nm with a 1 nm slit for excitation and emission.

Time-resolved photoluminescence (TR-PL) measurements were performed by exciting the samples with 200 fs long pulses delivered by an optical parametric amplifier (Light Conversion TOPAS-C, Vilnius, Lithuania) pumped by a regenerative Ti:Sapphire amplifier (Coherent Libra-HE, Santa Clara, CA, USA). The repetition frequency was 1 kHz and the PL signal was recovered by a streak camera (Hamamatsu C10910, Hamamatsu City, Japan) equipped with a grating spectrometer (Princeton Instruments Acton SpectraPro SP-2300, Trenton, NJ, USA). Samples were excited in the front face mode and proper optical filters were applied when needed.

Transmission Electron Microscopy (TEM) Images were collected by a Jeol JEM 1400 Plus (Tokyo, Japan).

All the quantum-chemistry calculations were performed by using the Gaussian 16 suite of programs [55]. For all the structures simulated, we performed a geometry optimization down to the self-consistent field (SCF) energy of each system by means of DFT calculations carried out at a B3LYP/6-311G(d,p) [56,57] level of theory. To account for the interaction of simulated structures with water, exploited both as solvent in the synthesis and dispersion medium for the analysis (Vide Infra), the self-consistent reaction field model was considered to include the solvation effects. The dielectric solvent was simulated through the polarizable continuum model calculation within the integral equation formalism (IEFPCM) [58]. No imaginary frequencies were calculated for all the optimized ground state structures in the vibrational spectra, thus assuring that the simulated structures were real energy minima. 

Optical absorption transitions in the UV-visible spectral range were simulated by TD-DFT calculations at the same level of theory. Vertical energy transitions from the ground state configuration were calculated with the solvent environment (water) fixed as in the ground state. 

## 3. Results

### 3.1. Experimental Results

**Figure 1:**
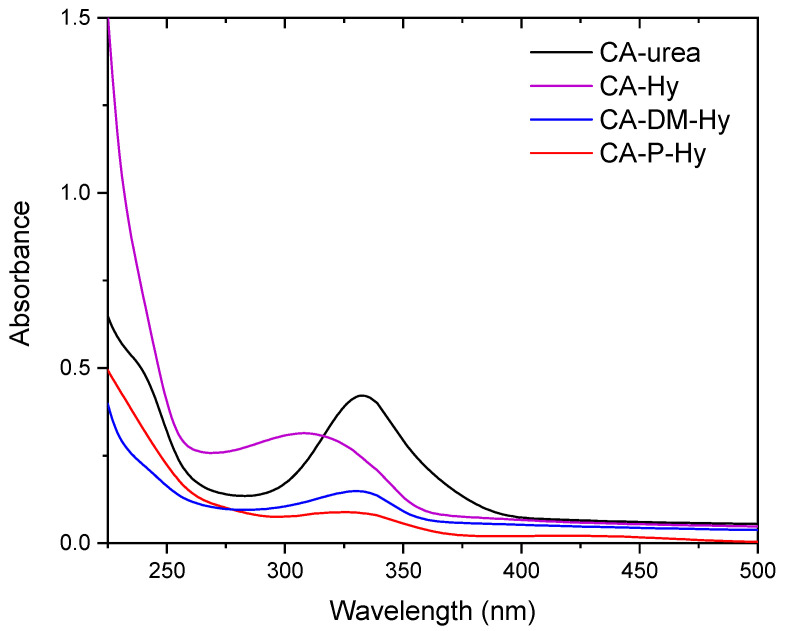
Absorption spectra of the hydrazine-based compounds compared to urea-derived carbon dots (CDs).

In order to verify the formation of CD-like structures, from hydrothermal treatment of citric acid and hydrazine compounds, detailed optical analysis was performed. Comparing the absorption spectrum of the hydrazine precursors and the obtained products, the observation of new and different bands indicates the formation of new compounds (Figure S1). Whilst the precursors have absorption in the far UV always below 300 nm, the formation of the band at about 330–350 nm is a distinctive signal of the possible formation of CDs. Indeed, in Figure 1, the absorption spectra of all samples present a shoulder in the UV region at 225–250 nm, which is generally ascribed to the π–π* transition of aromatic carbon that is supposed to be present in the CD core structure. An absorption peak at around 330 nm can be observed in all samples, except for CA-Hy, whose peak is at about 310 nm. This peak in the near UV region is typically attributed to the n–π* transition of N or O containing structures. These assignments are well confirmed by the computational results here reported (see computational results). The intensity of the 350 nm peak is higher in urea-sample than in hydrazine ones, suggesting the formation of a larger set of absorbing centers and possibly higher emission performances while, among the others, the sample derived from hydrazine hydrate shows the more intense peak (all the spectra were acquired considering the same amount of product dispersed in water).

PL measurements of precursors were also gathered to further clarify the effective formation of the products. The signal collected from precursors was weak, peaking in the near UV spectral range and several orders of magnitude lower than the respective CA and nitrogen sources products, thus confirming that PL properties are ascribed only to the formation of new luminescent products.

**Figure 2:**
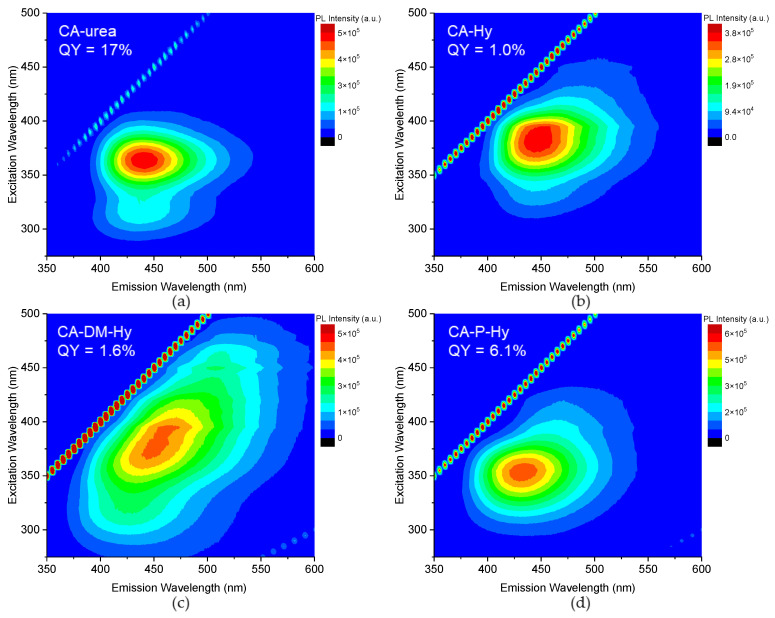
Excitation–emission maps of our reference CA-urea (**a**) compared to CA-Hy (**b**), CA-DM-Hy (**c**) and CA-P-Hy (**d**).

A complete overview of photoluminescence properties of the samples can be obtained through excitation-emission maps in which is possible to analyze the intensity of the emission at different excitation wavelengths (Figure 2).

Despite the low QY, estimated using as reference an ethanol solution of Coumarin 120 (C120) with spectral features close to our investigated samples (absorption peak at 350 nm, emission at 430 nm, QY = 0.56) [59], PL emission of all products is characterized by an excitation-dependent behavior, which is typical of CDs, indicating the formation of more than one excitation channel and not just a single molecule.

**Figure 3:**
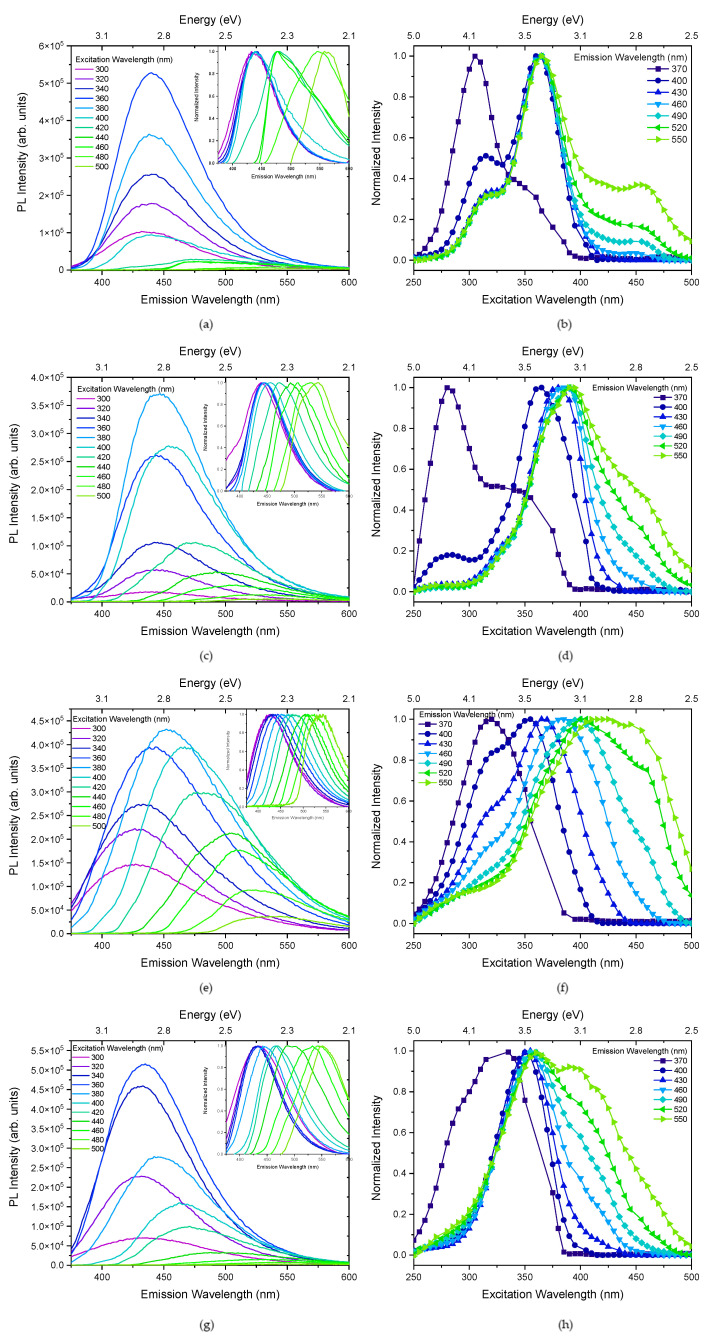
PL and normalized PL (inset) on the left column and normalized PL excitation (PLE) on the right one of CA-urea (**a**,**b**), CA-Hy (**c**,**d**), CA-DM-Hy (**e**,**f**) and CA-P-Hy (**g**,**h**).

Extracting the PL and excitation of PL (PLE) graphs (Figure 3), it is possible to notice that, despite the quite similar absorption spectra, the samples show many differences one to another. The reference derived from urea (Figure 3a,b) has the highest QY (17%) and presents basically three excitation channels: those around 310 and 360 nm which both brought to the emission at 440 nm and another oat about 460 nm that led to a weak contribution in the green region. Previous studies attributed these optical features to the presence of citrazinic acid and citrazinic amide in the structure of CD [11,60].

CA-Hy (Figure 3c,d) has the highest luminescent emission at 450 nm, with the main excitation channel centered at about 380 nm. Even in this case we can distinguish two more excitation channels: one at 290 nm, responsible for the near UV emission, and another one that arises at above 490 nm, which brought a green contribution. Overall, even if the sample presents a low QY (1.0%), it does show a clear tunable emissive behavior.

A clearer example of this peculiar characteristic is shown in CA-DM-Hy (Figure 3e,f), that not only presents an interesting broad emission centered around 455 nm under an excitation of 380 nm but also a great tunability under increasing excitation. Indeed, a slight change in the excitation results in an appreciable shift of emission, a property that makes this product potentially adaptable for a wide range of applications despite the low QY (1.6%).

Finally, CA-P-Hy (Figure 3g,h) displays the highest QY among the hydrazine products (6.1%) and the main emission band centered at 440 nm, resulting from the 350 nm excitation, represents the main contribution, paired with a blue excitation above 410 nm accountable for the emission at longer wavelengths. 

In all these cases, the evident tunability of the optical properties let us to suppose that the formation of hydrazine related CDs was of a different nature as compared to CA-urea sample.

**Figure 4:**
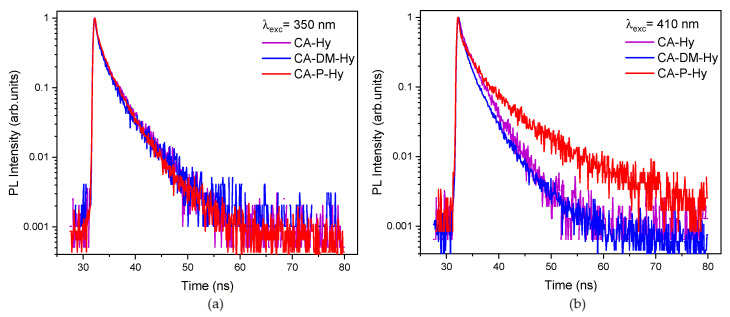
TR-PL decays of a sample excited at 350 nm (**a**) and 410 nm (**b**).

TR-PL measurements performed at 350 nm and 410 nm (Figure 4) confirm the presence of multiple emission channels as displayed by the non-single exponential profile of decay time investigated in the 50 ns time window (except for CA-urea which was measured in the 100 ns time window, Figure S2).

The average decay times were estimated through a multi exponential fit with two or three decays according to the sample under exam (Figure S2, Table 1). In all the cases, a sub-nanosecond decay time close to the time resolution of 0.4 ns (estimated by means of the signal from 10% to 90% risetime) has been reported. The average time was evaluated calculating the fractional contribution of each fitted decay time (Equation (S1)). All hydrazine composites excited in the 350 nm region show a decay time of about 2.6 nm. which let us imagine that the source of this emission could be the same in all samples. Instead, in the urea sample, the estimated lifetime is 5.6 ns, longer than in the other products, a value close to the one reported in literature [61,62,63]. Exciting the samples at higher wavelength, the average lifetime is the same in CA-Hy and CA-DM-Hy, but it definitely increases in CA-P-Hy with the presence of a longer time of 8.8 ns and reaching an average lifetime of 5.1 ns, close to that of CA-urea of 4.6 ns.

**Figure 5:**
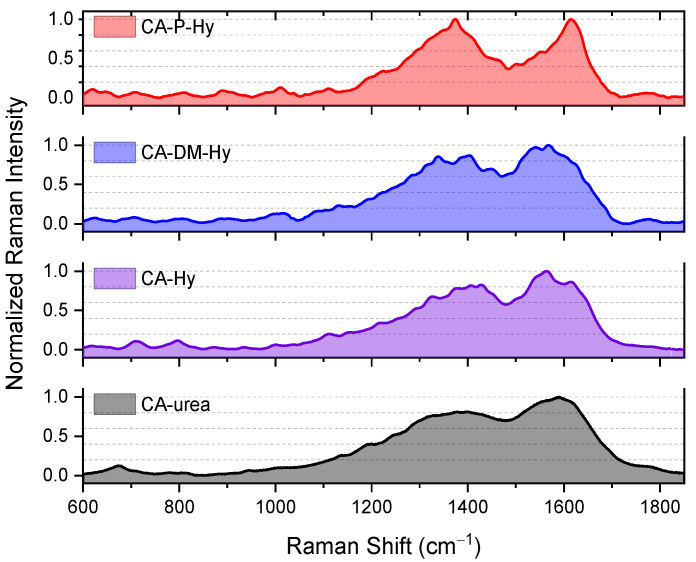
Raman spectra of the hydrazine-based compounds compared to urea-derived CDs.

Moreover, Raman analysis suggests the formation of CD-like structures. Raman spectra were collected in the 600–1850 cm^−1^ region using SERS supports which allow the enhancement of the vibration signals, reducing the fluorescence of the samples (Figure 5). All samples present two major peaks centered at about 1360 and 1580 cm^−1^, which are generally ascribed to sp^3^ and sp^2^ hybridized carbon structures and are often reported in CD literature [4,13,36,64]. Indeed, the so-called D band at 1360 cm^−1^ is associated with disordered carbon systems, whilst the G band at 1580 cm^−1^ is typical of graphite and consequently is related to graphitic morphology. For evaluating the relative contribution to the structure of the graphitic core state and the disordered surface state, it is useful to calculate the ratio of these bands. Roughly considering the height of the peaks, the highest level of disorder was reported for the CA-P-Hy sample (I_D_/I_G_ ≈ 1), which suggests that it is a poor graphitic system. Slightly lower values are associated to the other hydrazine-based samples (I_D_/I_G_ ≈ 0.8–0.9) which present similar spectra, only with small differences in the D band multiple peaks due to the high sensitivity of SERS supports. Even the urea-derived reference sample shows an analogous disorder/order ratio value (I_D_/I_G_ ≈ 0.8) along with the most smoothed spectrum with little presence of defined peaks, which could be due to the completed formation of CDs. Smaller vibrational peaks gathered upon the larger signal of the two main bands are present and well above the sensitivity limit of the measurement. These vibrational findings have also been confirmed by simulated results (see computational results and Figure S3).

TEM images of hydrazine-derived CDs are reported in the Supplementary Materials (Figure S4). The images show that the performed synthesis produced rounded nanoparticles with mean size of about 4–5 nm for the CA-Hy and CA-DM-Hy samples and about 10–13 nm for the CA-P-Hy ones. 

### 3.2. Computational Results

**Scheme 1:**
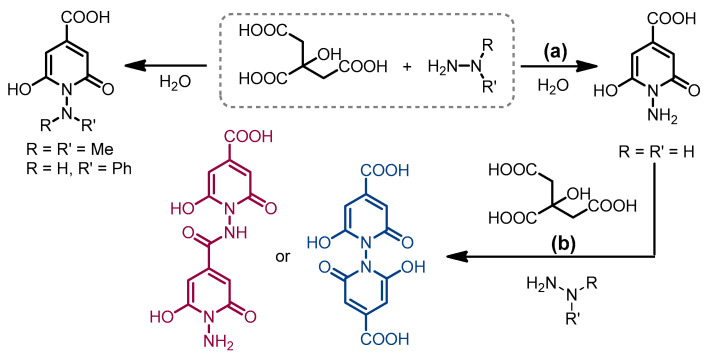
Reaction scheme of citric acid and substituted hydrazine molecules. (**a**) In the case of hydrated hydrazine, the reaction can further proceed, leading to di-pyridinone structures (**b**).

The possible mechanism of reaction among the two precursors during hydrothermal synthesis is reported in the following scheme (Scheme 1), where citric acid and hydrazine react to produce a pyridinone molecule—by means of condensation and dehydration reactions—with different possible substituents depending on the starting hydrazine molecule precursor (Scheme 1, path a). Depending on R and R’, we can obtain a simple 1-aminopyridin-2(1H)-one (Hy-CD), a mono-methyl 1-(methylamino)pyridin-2(1H)-one (MM-Hy-CD), a 1-(dimethylamino)pyridin-2(1H)-one (DM-Hy-CD) or a 1-(phenylamino)pyridin-2(1H)-one (P-Hy-CD). In the simulations, we also considered the formation of MM-Hy-CD for completeness’ sake, even though it is not experimentally expected considering the exploited precursors. In the case of simple hydrazine, we can also hypothesize that the reaction might evolve through the dimerization (or oligomers formation) as described in the Scheme 1 path b, once more by means of condensation and dehydration reactions, leading to di-pyridinone structures. The formation of the two different products, called Hy-2-CD and Hy-3-CD in the following, is represented in the Scheme 1b.

We calculated the optimized ground state structures of the possible pyridinone and di-pyridinone molecules (Hy-CD, Hy-2-CD and Hy-3-CD) and simulated their optical absorption spectra in the UV-vis range. We also calculated the optical features of the substituted pyridinone structures (MM-Hy-CD, DM-Hy-CD and P-Hy-CD). Finally, for all the structures, we calculated the vibrational spectra, both IR and Raman.

**Figure 6:**
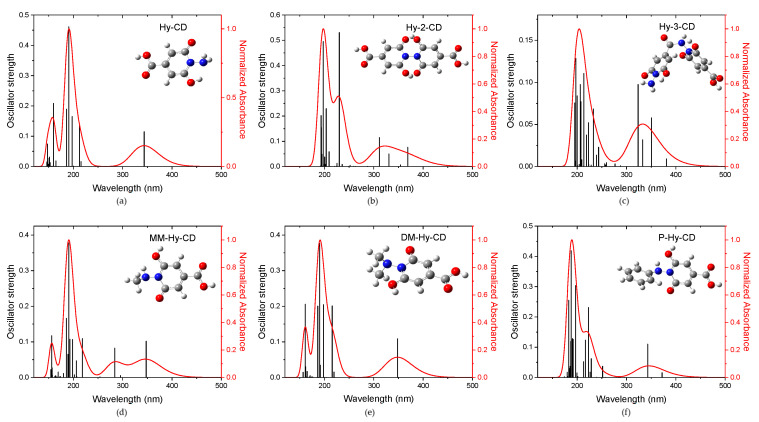
Simulated absorbance spectra and oscillator strength of pyridinone molecules. The insets report the ball-and-stick representation of the structures (H atom—white sphere, C atom—grey sphere, N atom—blue sphere, O atom—red sphere). (**a**) Hy-CD, (**b**) Hy-2-CD, (**c**) Hy-3-CD, (**d**) MM-Hy-CD, (**e**) DM-Hy-CD, (**f**) P-Hy-CD.

As reported in Figure 6a–c (top panel), all of the three molecules can provide transitions, accounting for an absorption band around 350 nm and a higher energy band in the 150–250 nm range. The normalized absorbance spectra (continuous red line in the figure) were simulated by assuming Gaussian bands of 0.33 eV half width at half height of the peaks centered at the transition energies of the first 20 calculated excited states (whose oscillator strength is reported as vertical black lines in the figure). The first absorbance band in the simplest Hy-CD mono-pyridinone structure is due to the single Highest Occupied Molecular Orbital (HOMO) to the Lowest Unoccupied Molecular Orbital (LUMO) transition, located at 344 nm. The di-pyridinone structures show a larger absorbance band around 350 nm with a red-shifted HOMO–LUMO gap, at 369 and 381 nm for the Hy-2-CD and Hy-3-CD molecules, respectively. Concerning the substituted pyridinone structures, they also provide similar absorbance features (Figure 6d–f, bottom panel) with a HOMO–LUMO gap at 348 nm, 350 and 372 nm for MM-Hy-CD, DM-Hy-CD and P-Hy-CD, respectively. We note that the MM-Hy-CD structure presents a second excitation band around 300 nm, not observed in the other structures, and the P-Hy-CD structure displays the most red-shifted optical transition among the substituted pyridinone structures.

The computed vibrational features of the hydrazine precursors and those of the pyridinone structure are compared in Figure S5 in the 1250–1700 cm^−1^ band, where the G and D band were experimentally observed. None of the precursor vibrational modes were present in both the 1350–1400 and 1550–1600 cm^−1^ regions, whilst the pyridinone structure shows two bands at 1400 and 1580 cm^−1^.

**Figure 7:**
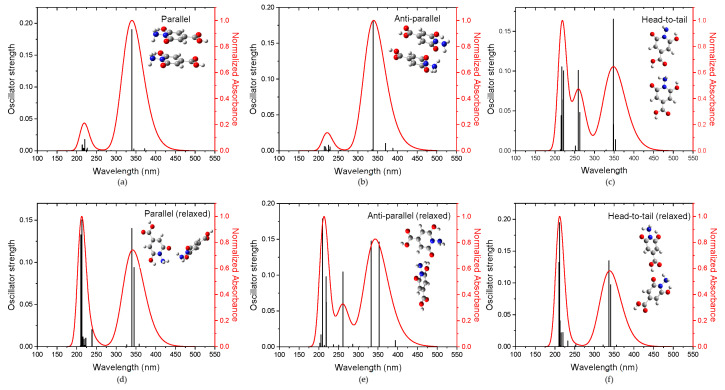
Simulated absorbance spectra and oscillator strength of parallel, anti-parallel and head-to-tail dimers for fixed geometries (**a**–**c**) and for the corresponding relaxed geometries (**d**–**f**). The insets report the ball-and-stick representation of the structures (H atom—white sphere, C atom—grey sphere, N atom—blue sphere, O atom—red sphere).

To further explore the scenario, we considered for the Hy-CD case the formation of some typical aggregates already proposed for the CZA and IPCA systems [65,66,67,68,69], namely the parallel and anti-parallel stack dimers and the head-to-tail in plane dimer. We calculated the absorption spectra for the aggregate systems at a fixed distance between the monomer units, namely at 0.366 nm for the stack dimers and at 0.288 for the heat-to-tail dimers, assuming the same geometries of the parent CZA system. We also let the dimers relax and calculate the absorption spectra in the optimized relaxed configurations. The spectra are reported in Figure 7: all the structures display a large absorption band in the 300–400 nm range, with HOMO–LUMO transition at 377 and 358 nm for the fixed and relaxed parallel stack dimers, at 389 and 394 nm for the fixed and relaxed anti-parallel stack dimers and at 354 and 356 nm for the fixed and relaxed head-to-tail dimer.

**Scheme 2:**
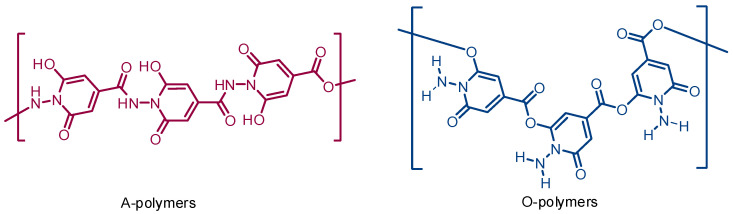
Possible formation of polymeric chains for the Hy-CD case.

Finally, to look for larger red shifts, we considered the formation of some possible polymers. Indeed, one can hypothesize that, during the carbonatation process, some polymeric structure is formed, with the release of water. Among the possible choices, we calculated two potential polymer structures. For the simple Hy-CD system and the substituted di-methyl and phenyl systems we hypothesized the polymer chains that can be obtained when considering the reaction between the carboxyl group of one monomer and the hydroxyl group of another, as represented in Scheme 2 for the case of the Hy-CD structure (this class of polymers will be indicated as O-Polymer in the following). We considered chains of two, three and four units. A second possibility is to assume the formation of a peptide bond to form the polymer chain, thus considering the reaction of the carboxyl group of one monomer with the amide group of the other (A-Polymer). This bonding is the same that we have in the Hy-3-CD structure, which can be also considered as the first member of the A-Polymer family for the un-substituted hydrazine case (Hy-CD). The formation of the A-Polymer class was not considered for the substituted hydrazine precursors, since we expect that steric volume of the substituents would reduce the probability of formation of A-Polymer in those cases. Although the reactivity of the amine group would favour the formation of A-Polymers, O-Polymers could also be expected, since the computed difference of formation energy among the two classes for the case of two units’ chain was estimated as 4.83 kcal/mol (0.21 eV). 

**Figure 8:**
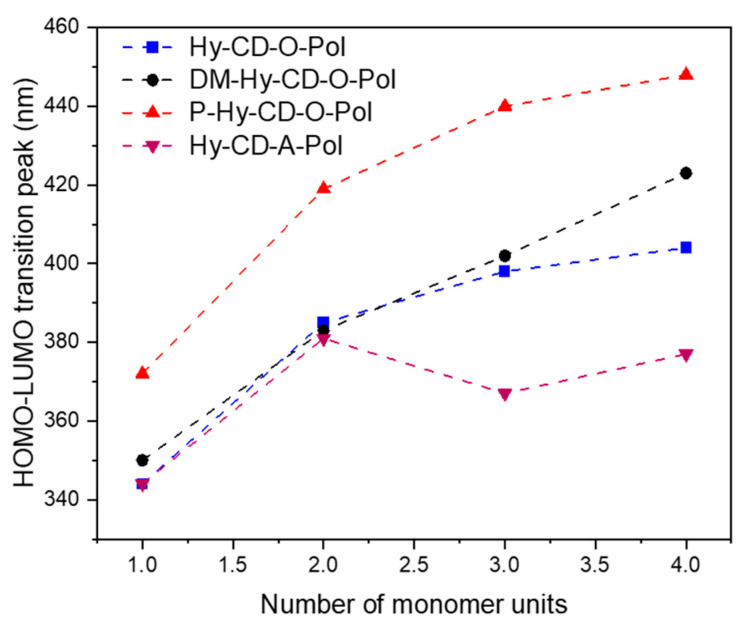
HOMO–LUMO transition wavelength as a function of the number of monomer units for the two classes of hypothesized polymers (lines are guides for the eyes).

The calculated optimized ground state polymeric structures are reported in the Supplementary Materials, together with the absorbance spectra of those structures (Figure S6). To collect the general trend, in Figure 8 we report the position of the HOMO–LUMO transition as a function of the number of monomer units for both the O-Polymer and A-Polymer classes. As we can see, the longer the chain the larger the red shift, a sort of saturation effect in the red shifting being recorded for all the structures except the DM-Hy-CD ones.

**Figure 9:**
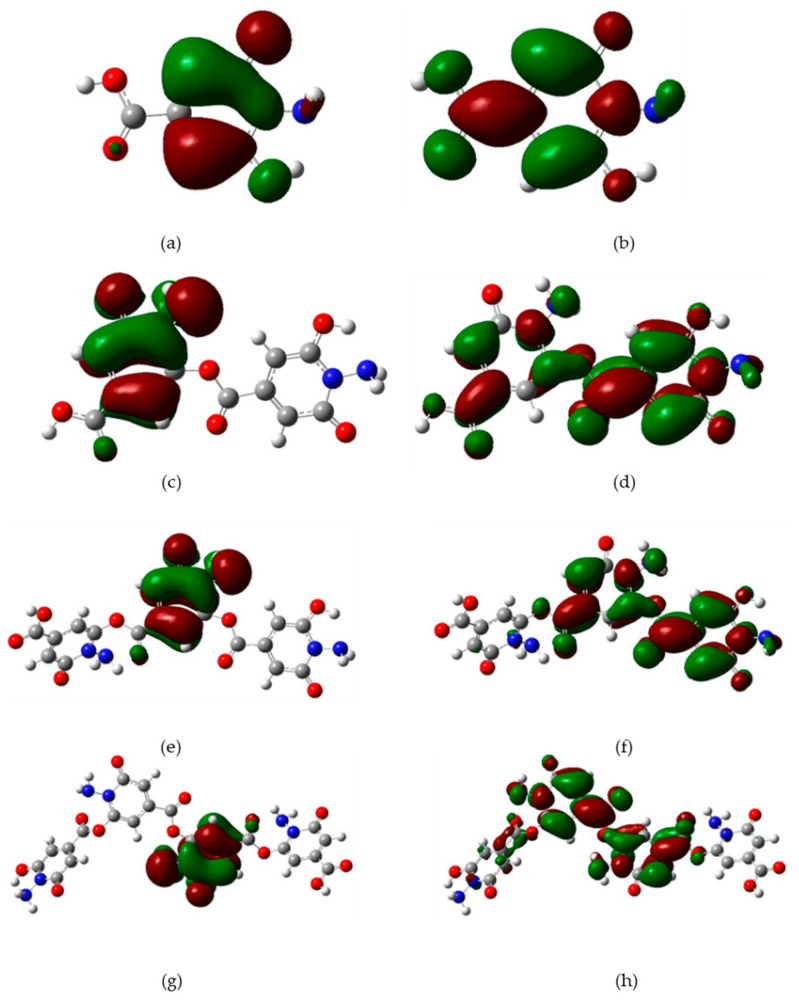
MOs of the HOMO–LUMO states (HOMO on the left and LUMO on the right) for the Hy-CD system (**a**,**b**) and its O-Polymer derived: Hy-CD-O-Pol-2 (**c**,**d**), Hy-CD-O-Pol-3 (**e**,**f**), Hy-CD-O-Pol-4 (**g**,**h**). The isocontour value is 0.02 au (H atom—white sphere, C atom—grey sphere, N atom—blue sphere, O atom—red sphere).

Finally, we calculated the molecular orbitals (MOs) for the HOMO and LUMO states for all the simulated structures. In Figure 9 we report, as an example, the MOs for the Hy-CD case and its O-polymer derivatives. The MOs of the other structures are reported in the Supplementary Materials (Figures S7 and S8). The reported data depict a n–π* transition for the whole set of structures, with the electronic charge distributed upon a single unit in the HOMO state and spread upon two units in the LUMO state, partially extending through the chain bonding for the cases with three and four units.

## 4. Discussion

The optical features of CDs are, in general, ascribed to three main mechanisms: the quantum confinement effects, related to the formation of graphitic core, the presence of molecular species or the formation of surface centers related to the surface functionalization groups [9,10]. The formation of fluorescent molecules that can act as seed for the CD enucleation or could be incorporated within the carbon network is the most accredited model in the case of bottom-up synthesis. The target of this study is the proof of concept of the formation of hydrazine-related CDs. The whole set of experimentally observed features here reported calls for the formation of CDs: the rounded nanoparticle observed by means of TEM (mean size 5–12 nm), the measured D and G vibrational bands, the absorption band around 350 nm, the excitation dependent emission spectra and the non-single exponential decay times. As already proposed for parent CA derived systems, those features are related to the formation of some specific molecule, as the IPCA and CZA molecules reported in those cases [11,28,65,66]. Starting from this consideration, we hypothesized that the pyridinone molecules produced during the synthesis of CA and hydrazine, their aggregated forms or some polymeric structure derived by further reactions of the monomer units can be incorporated into the structure of the CDs and produce the optical features experimentally recorded. Indeed, if we consider the case of IPCA, a reaction of cyclization between CA and ethylene-diamine was hypothesized to produce the molecule that acts as a seed for the formation of the CDs and cause, with its aggregates, the excitation dependent emission features recorded in those systems [28,67,68]. In a similar way, we recently studied the formation of CZA in the synthesis of CA and urea (the reference system in the present study) and we demonstrated that the formation of various ionic species and different aggregates can explain the optical features of citric acid derived CDs [65,66,69,70]. If we assumed the same reaction paths reported for CZA and IPCA, more than one product could be embedded during the CD formation. To also test this hypothesis in the present case, we calculated the vibrational and optical absorption spectra of different possible structures that we expect could be formed during the hydrothermal synthesis and compared the simulations to the experimental results. We first discuss Raman properties. Raman spectroscopy is largely applied to explore CD structures and in particular to evaluate the sp^2^/sp^3^ ratio, which is an important parameter also concerning the attribution of the emission properties, especially when applying the core-shell model [63,71]. We considered the computed vibrational features of the hydrazine precursors and compared them to the ones of the pyridinone structure. As reported in Figure S5, the formation of these structures produces a few vibrational modes in the 1250–1700 cm^−1^ range, that of the experimentally observed D and G band (Figure 5 and Figure S3). Among the others, the two vibrational modes at 1400 and 1580 cm^−1^ are related to symmetric and asymmetric stretching vibrations of the atoms of the pyridinone ring. Similar results were obtained for all the simulated structures with the pyridinone ring. The precursors, on the contrary, did not show paired vibrations that could be referred to the G and D band. Moreover, the experimental Raman spectra are very similar to the reference CD sample. The presence of some overimposed fine structures can be related to the presence of polyaromatic hydrocarbon structures, which could also give similar results [72]. These findings support, from one side, the hypothesis of the formation of the pyridinone structure, and from the other, the possible formation of CDs in the synthesis of CA and hydrazine compounds. Looking at the optical features, we considered both a single pyridinone molecule produced by the cyclization of citric acid and hydrazine (Hy-CD) and the formation of di-pyridinone structures obtained by the reaction of the first system with residual citric acid and hydrazine in the solution (Hy-2-CD and Hy-3-CD). The simulated spectra (Figure 6) display two bands in the near and far UV in good agreement with previous calculated results on parent structures (CZA and IPCA) and in good agreement with the reported experimental absorption measurements (Figure 1). Concerning the absorption band in the 300–350 nm range, typically ascribed to a n–π* transition in CDs, as also confirmed by our calculated MOs for all the considered structures (Figure 9, Figures S7 and S8), we note that the present experimental results and the ones reported in the literature on similar hydrazine-related CDs show a peak at about 330 nm, thus preferentially supporting the hypothesis of the single pyridinone molecule instead of the two red-shifted di-pyridinone structures. However, the width of the band could indicate the presence of more than one pyridinone species, possibly including the di-pyridinone structures or other systems such as aggregates or polymer chains. Indeed, accounting for the larger sensitivity of the photoluminescence measurements, we should also consider that the excitation emission maps here reported (Figure 2) display excitation transitions in the 330–450 nm range for the CA-Hy samples, in the 300–450 nm range for the CA-DM-Hy and in the 300–425 nm range for the CA-P-Hy. These data fully support the absorbance spectra of the simulated structures and even call for more complex structures to account for such a large excitation band, in particular for the CA-DM-Hy case. These results also highlight the optical difference between hydrothermally produced urea-derived CDs and these hydrazines compounds, showing that the latter are characterized by a higher tunability with a consequently larger contribution of the emission in the green region. In addition, the non-single exponential decays recorded for the different systems also require the formation of at least two or three different emitting centers. For these reasons, we first considered the formation of a few model aggregates, the parallel and anti-parallel stack dimers, obtained from the parallel or anti parallel superimposition of two monomer units of Hy-CD, and the head-to-tail dimer, where the two monomer units lie on the same plane. Those aggregates were selected according to the results already reported for CZA and IPCA. Although the absorption spectra simulated for all the dimers at fixed distance or after relaxing the system are in the expected 300–400 nm range and could contribute to the experimentally observed absorption band in that range, we still needed to find some structure able to provide redder transitions. In addition, we noted that the relaxed geometries are quite far from those assumed by comparison with the CZA system. Thus, instead of collecting other aggregates’ forms, as already proposed for the CZA case [65], where the broadening of the absorption and excitation bands was smaller than in the present case, we considered the formation of small polymer chains of two, three and four units and the possibility of two different polymerization processes, depending on the chemical groups interested by the reaction. We considered up to four units because it should be noted that the longer the chain, the larger the distance between the two ends of the chain and the distortion of the optimized geometry; when four units are considered, a distance of about 2–3 nm is achieved in the simulated structures, in agreement with the typical average diameter of Carbon Dots. As reported in Figure 8 and in the Supplementary Materials, we calculated, in general, a red shift of the whole spectrum for all the simulated structures, with the HOMO–LUMO transition peak shifting up to 460 nm for the larger phenyl-hydrazine polymer considered. These findings support the hypothesis that beside the presence of more species, the formation of a small polymeric chain can explain the large excitation spectra observed in the experimental data and the possible formation of different emitting centers. We also note that whilst the red shift of the HOMO–LUMO peak seems to converge to a wavelength value in the blue range for the hydrazine and phenylhydrazine systems, this is not the case for the di-methyl one, where the red shift does not show, at least for the length of the chain investigated, the above-mentioned saturation effect. This consideration agrees well with the observed larger excitation band for these CDs and calls for the presence of even longer polymer chains or more complex structures.

## 5. Conclusions

The aim of this work was to investigate the formation of possible fluorescent N–N heterocyclic structures which could represent the seed for the synthesis of CDs and the origin of their emission properties by using, as precursors, N–N bonds containing hydrazines. The experimental results suggest the formation of compounds which present optical characteristics that could be compatible with such structures, as reported in similar synthetic treatment of CA and ammine sources, giving aromatic products such as IPCA and CZA. For instance, Raman and UV-Vis spectra assess the formation of aromatic products that are different from the original, not cyclic, precursors. Moreover, excitation-emission maps underline that the obtained products show different degrees of emission tunability, in all cases with an emissive region larger than the urea-based CDs. These findings represent a sign of evident non single molecule nature that is also confirmed by multi exponential decay spectra. Despite the low QY that could be increased by high standard sample purification, our products display undoubtedly interesting optical properties. The presence of multiple emission channels can be explained by the presence of different stages of the reaction leading to the formation of pyridinone-based structures. Indeed, since similar reactions were reported for parent fluorophores such as CZA and IPCA that have the tendency to form tautomers and can co-exist in different aggregation states, the same trend is expected to occur in the present case. In order to verify this hypothesis, we simulated the vibrational and optical absorption spectra of possible assembled structures, considering the formation of single and di-pyridinone compounds, also with different substituents depending on the hydrazine precursor. The computed Raman spectra confirmed the formation of the heterocycle structure, also supporting the observed D and G vibrational bands. Although those structures do show a HOMO–LUMO transition in the 300–400 nm range in good agreement with the experimental absorption spectra, we also considered more complicated structures to explain the large excitation band experimentally recorded. Besides some dimers, which provided small broadening of the main absorption band, we considered the formation of short oligomers that made up to four monomer units. The HOMO–LUMO gap increases as the number of monomer units increases, showing a saturation effect for all the structures except the di-methyl one. Indeed, this system presents the broader excitation pattern, suggesting the formation of even larger polymers. This proof of concept work suggests that N–N-doped Carbon Dots could be produced, starting from the formation of hydrazine derived heterocycle fluorophores. Along with TEM images, the assessed optical properties need to be further confirmed by advanced structural measurements such as XPS and NMR to definitely assess the formation of the pyridinone structures in this new family of CDs.

## Figures and Tables

**Table 1:** Fitting results of decay time data excited at 350 and 410 nm in the 50 ns window. A_i_ is the pre-exponential factor, τ_i_ is the lifetime, f_i_ is the fractional contribution and ⟨τ⟩ is the average lifetime (Equation (S1)).

**Sample**	$A_{1}$	$\tau_{1}$ (ns)	$A_{2}$	$\tau_{2}$ (ns)	$A_{3}$	$\tau_{3}$ (ns)	$f_{1}$	$f_{2}$	$f_{3}$	⟨τ⟩ (ns)
CA-urea@350 nm ^1^	1141	0.7	1025	3.1	566	8.2	0.09	0.37	0.54	5.6
CA-Hy@350 nm	1310	0.5	890	3.1	-	-	0.19	0.81	-	2.6
CA-DM-Hy@350 nm	659	0.6	384	3.1	-	-	0.24	0.76	-	2.5
CA-P-Hy@350 nm	1795	0.3	1890	1.4	1304	3.6	0.06	0.33	0.61	2.7
CA-urea@410 nm	1578	0.4	1139	2.4	608	6.8	0.09	0.36	0.55	4.6
CA-Hy@410 nm	795	0.8	836	3.0	-	-	0.20	0.80	-	2.6
CA-DM-Hy@410 nm	3119	0.4	3267	1.7	950	4.2	0.13	0.51	0.37	2.5
CA-P-Hy@410 nm	761	0.4	533	2.6	150	8.8	0.10	0.46	0.44	5.1

^1^ Analyzed in the 100 ns time window with time resolution of 0.7 ns.

## Data Availability

Data available on request due to privacy restrictions.

## Supplementary Materials

The following supporting information can be downloaded at: https://www.mdpi.com/article/10.3390/ma15041468/s1, Figure S1: Absorption of hydrazine precursors, Figure S2: Fitted decay times recorded under 350 nm and 410 nm excitation light, Equation (S1): Average lifetime formula, Figure S3: Comparison experimental–computational Raman vibrations of hydrazine hydrate sample, Figure S4: TEM images of CA-hydrazines compounds, Figure S5: Computed Raman vibrations of precursors and Hy-CD, Figure S6: Computed absorbance spectra of DM- and Ph-Hy-CD polymers, Figure S7: MOs of the HOMO–LUMO states for the DM-Hy-CD system and its O-Polymer derived system, Figure S8: MOs of the HOMO–LUMO states for the Ph-Hy-CD system and its O-Polymer derived system.
