# Supplementary material for: Towards N–N-Doped Carbon Dots: A Combined Computational and Experimental Investigation
Source: Materials (Basel). 2022 Feb 16;15(4):1468. doi: 10.3390/ma15041468 (PMC8880414; doi:10.3390/ma15041468)
Supplement: Supplementary file 1 [file materials-15-01468-s001.zip › materials-1560182-supplementary.pdf]

materials

MDPI

Towards N–N-doped Carbon Dots: a Combined Computational and Experimental Investigation

Chiara Olla [1]*, Stefania Porcu [1], Francesco Secci [2], Pier Carlo Ricci [1] and Carlo Maria Carbonaro [1,]*

[1] Department of Physics, University of Cagliari, Cittadella Universitaria, I-09042 Monserrato, Italy; chiara.olla@dsf.unica.it (C.O.); stefania.porcu@dsf.unica.it (S.P.); carlo.ricci@dsf.unica.it (P.C.R.); cm.carbonaro@dsf.unica.it (C.M.C.)

[2] Department of Chemistry and Geological Science, University of Cagliari, Cittadella Universitaria, I-09042 Monserrato, Italy; fsecci@unica.it (F.S.)

* Correspondence: chiara.olla@dsf.unica.it (C.O.); cm.carbonaro@dsf.unica.it (C.M.C.)

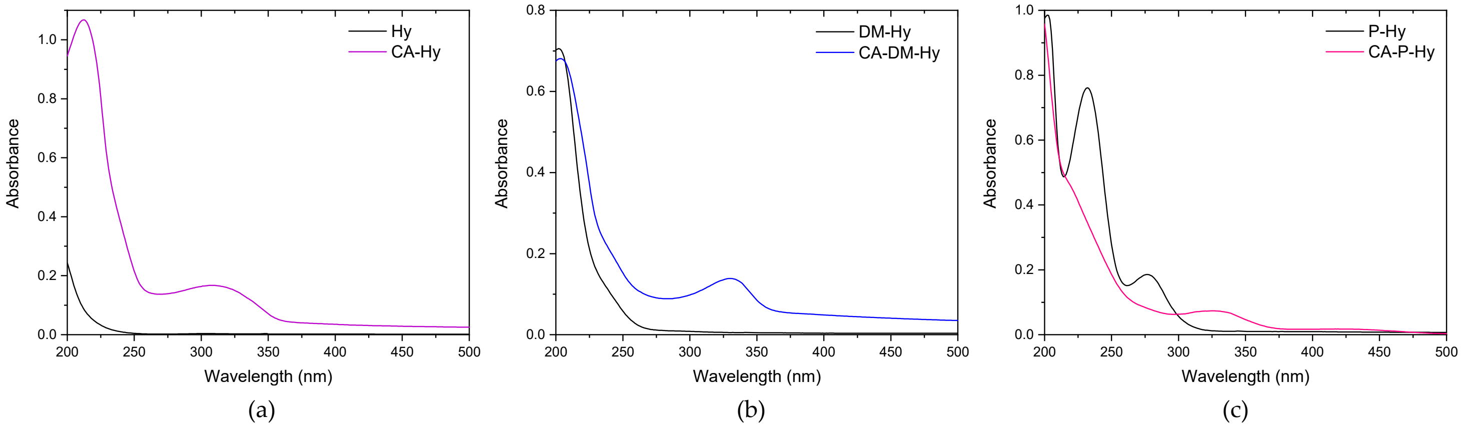

Figure S1. Absorption spectrum of hydrazine precursors compared to the corresponding product. (a) Hy and CA-Hy; (b) DM-Hy and CA-DM-Hy; (c) P-Hy and CA-P-Hy.

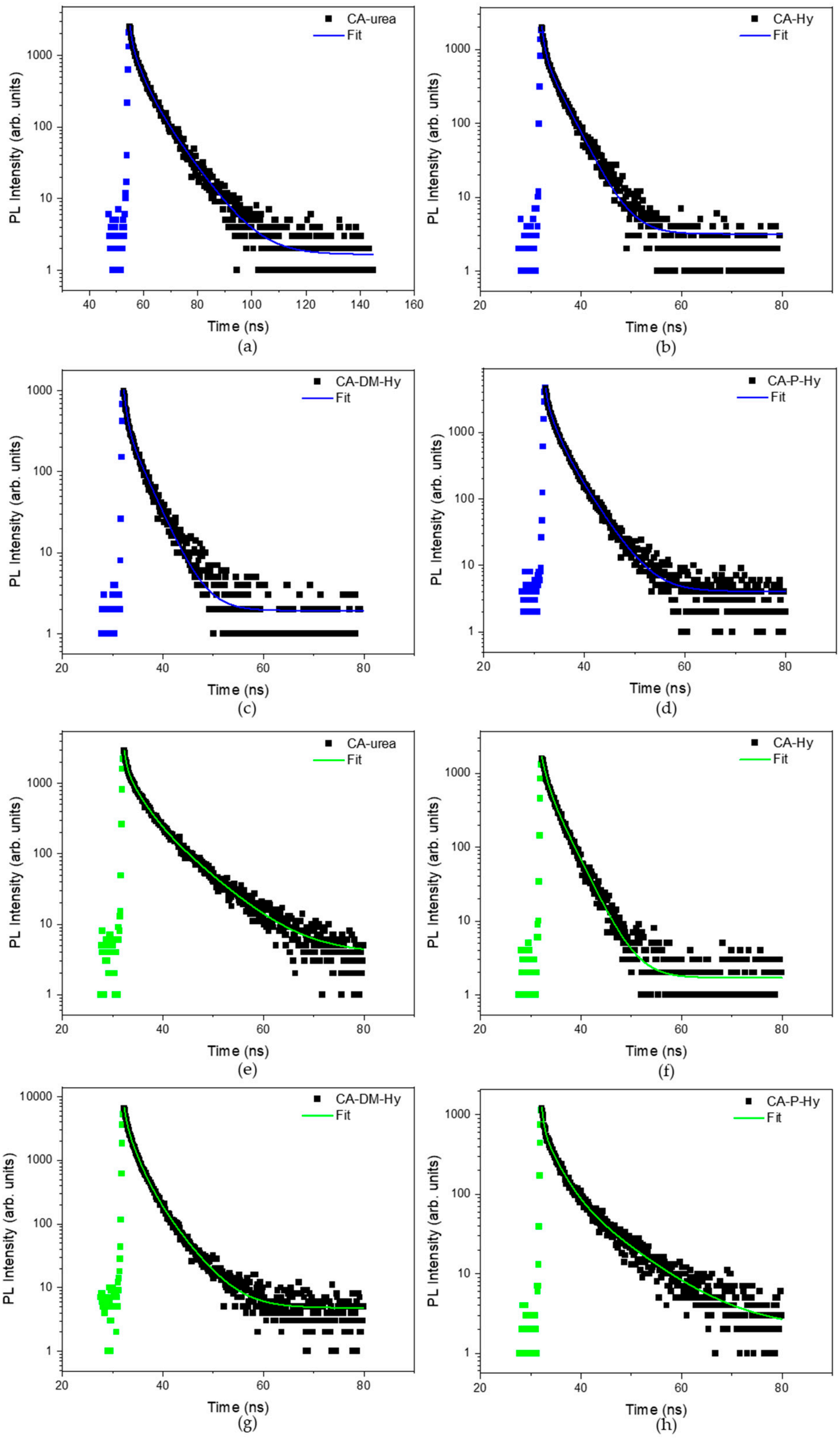

Figure S2. Decay times recorded under 350 nm (blue) and 410 nm (green) excitation light. (Risetime 0.7 in 100 ns, 0.4 in 50ns.) (**a**) CA-urea excited at 350 nm in 100 ns; (**b**) CA-Hy excited at 350 nm in 50 ns; (**c**) CA-DM-Hy excited at 350 nm in 50 ns; (**d**) CA-P-Hy excited at 350 nm in 50 ns; (**e**) CA-urea excited at 410 nm in 50 ns; (**f**) CA-Hy excited at 410 nm in 50 ns; (**g**) CA-DM-Hy excited at 410 nm in 50 ns; (**h**) CA-P-Hy excited at 410 nm in 50 ns.

Equation S1. The average lifetime was calculated as the weighted mean of the retrieved values[1].

$$\bar{\tau} = \sum_{i=1}^{n} \frac{A_i \tau_i^2}{A_i \tau_i} = \sum_{i=1}^{n} f_i \tau_i \quad (1)$$

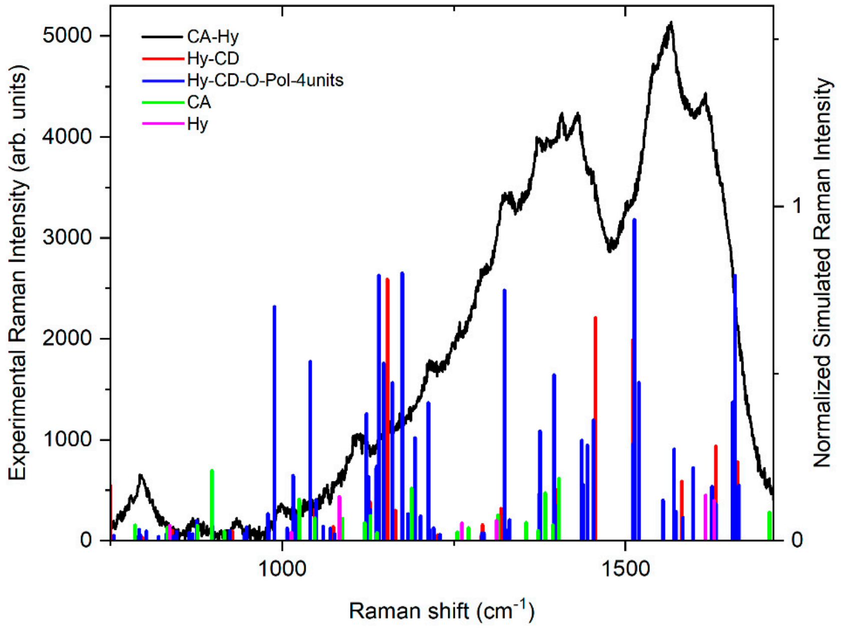

Figure S3. Comparison of experimental Raman spectrum of CA-Hy and computed Raman vibrations for precursors (CA and Hy) and possible Hy-CD structures.

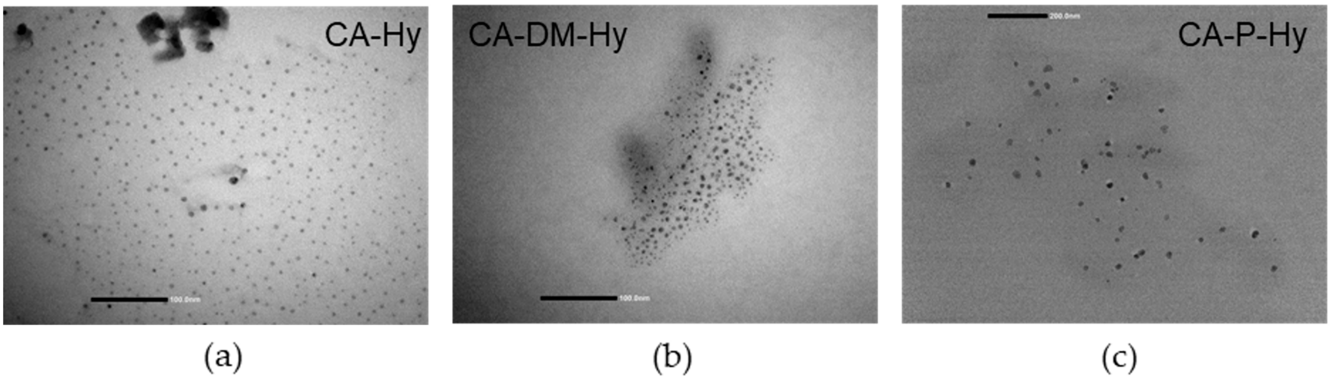

Figure S4. TEM images of CA-hydrazine compounds. (**a**) CA-Hy (100 nm scale bar); (**b**) CA-DM-Hy (100 nm scale bar); (**c**) CA-P-Hy (200 nm scale bar).

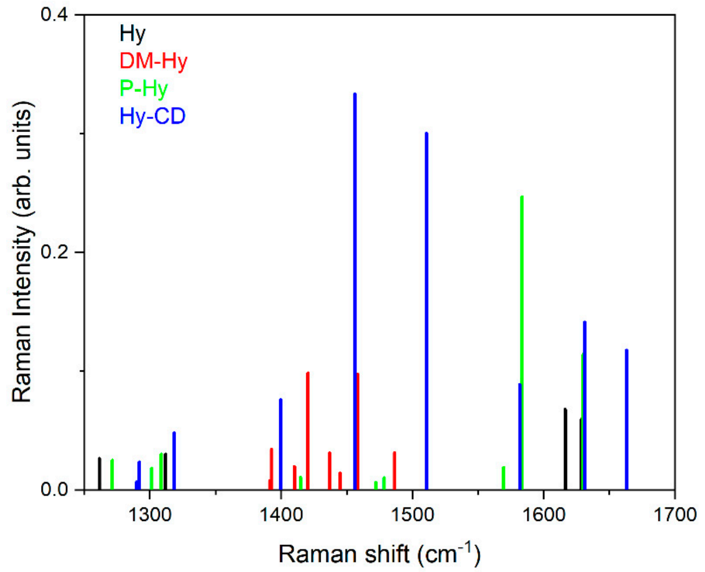

Figure S5. Computed Raman vibrations for hydrazine precursors and Hy-CD structure.

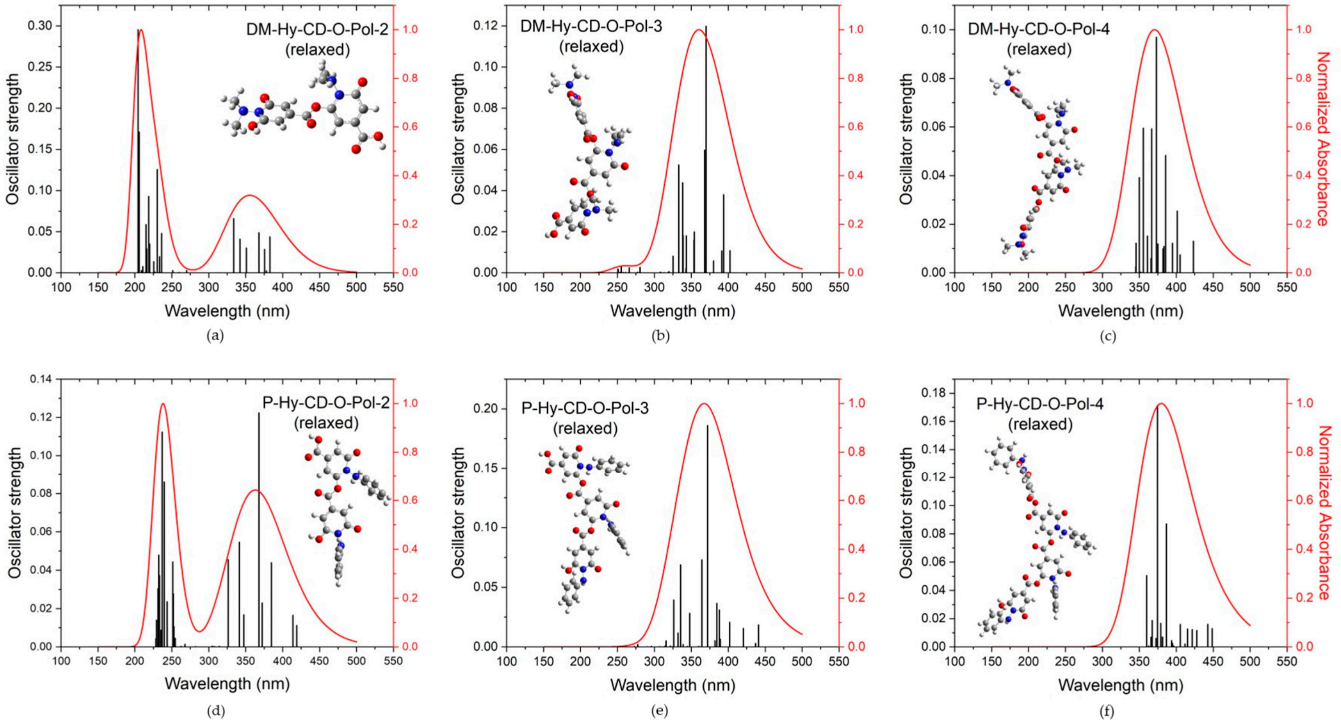

Figure S6. Simulated absorbance spectra and oscillator strength of DM- and P-Hy-CD polymers. The insets report the ball-and-stick representation of the structures (H atom—white sphere, C atom—grey sphere, N atom—blue sphere, O atom—red sphere). (a) DM-Hy-CD-O-Pol-2; (b) DM-Hy-CD-O-Pol-3; (c) DM-Hy-CD-O-Pol-4; (d) P-Hy-CD-O-Pol-2; (e) P-Hy-CD-O-Pol-3; (f) P-Hy-CD-O-Pol-4.

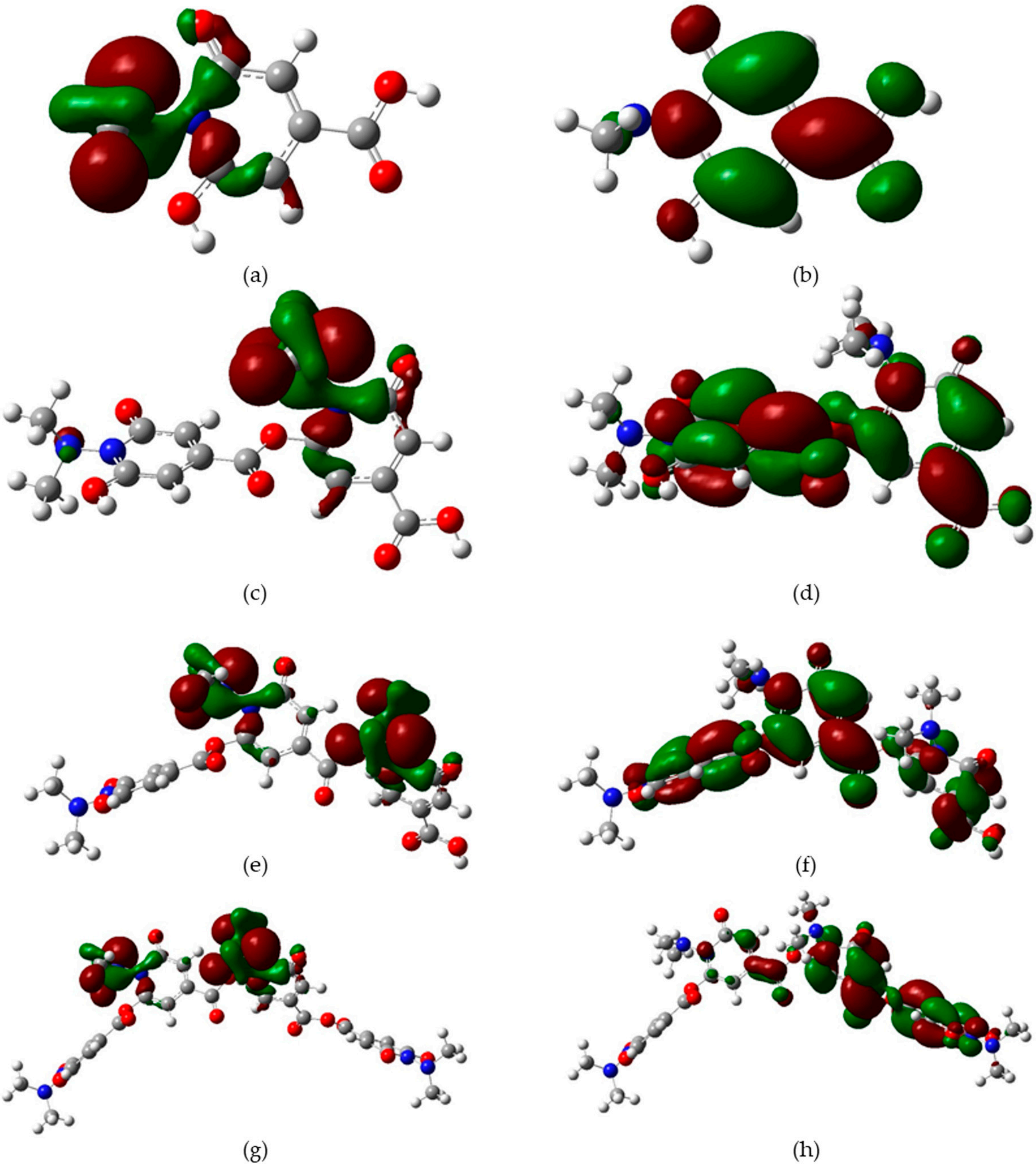

Figure S7. MOs of the HOMO–LUMO states (HOMO on the left and LUMO on the right) for the DM-Hy-CD system (**a,b**) and its O-Polymer derived system: DM-Hy-CD-O-Pol-2 (**c,d**), DM-Hy-CD-O-Pol-3 (e,f), DM-Hy-CD-O-Pol-4 (**g,h**). The isocontour value is 0.02 au (H atom—white sphere, C atom—grey sphere, N atom—blue sphere, O atom—red sphere).

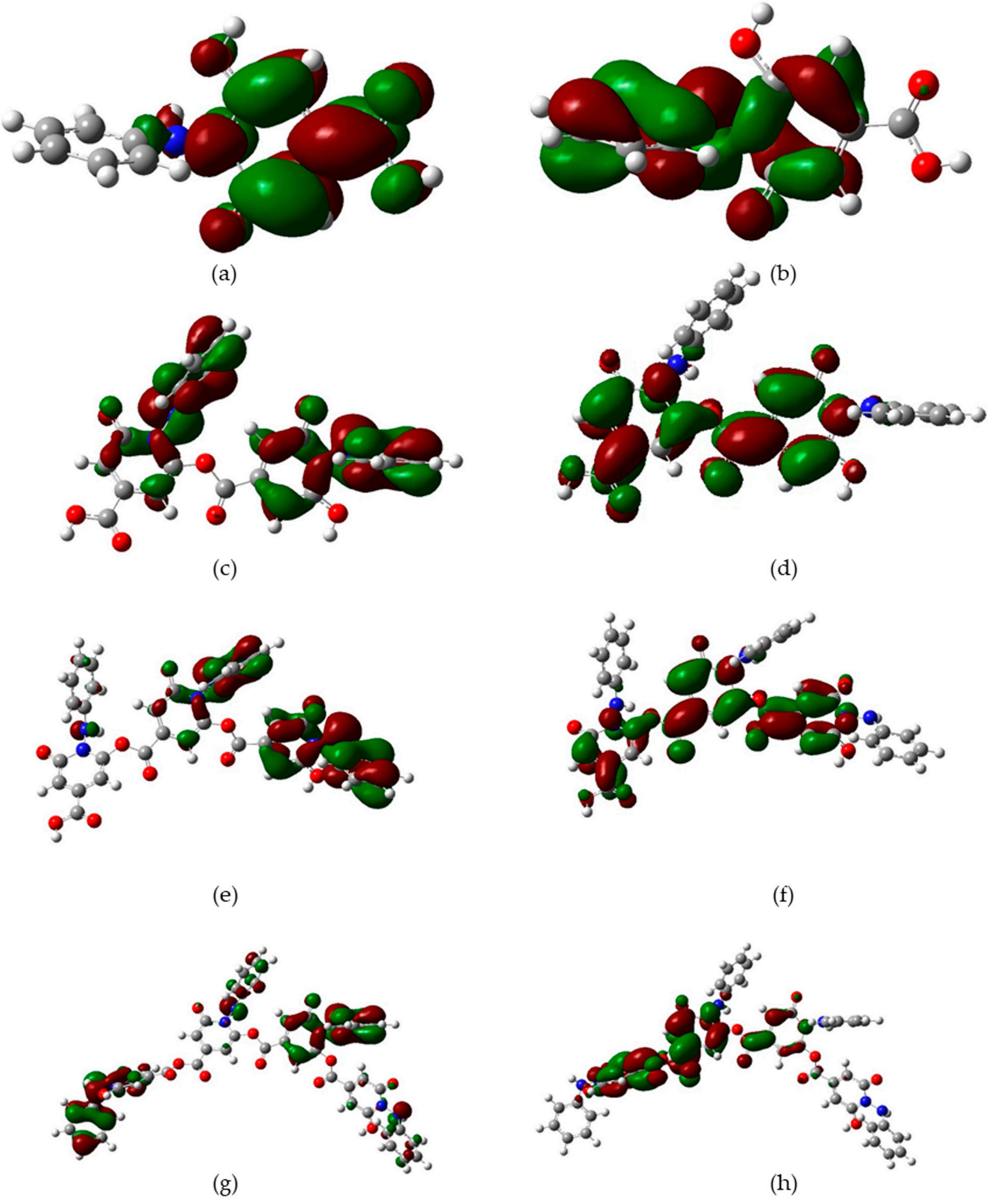

Figure S8. MOs of the HOMO–LUMO states (HOMO on the left and LUMO on the right) for the P-Hy-CD system (**a**,**b**) and its O-Polymer derived system: P-Hy-CD-O-Pol-2 (**c**,**d**), P-Hy-CD-O-Pol-3 (**e**,**f**), P-Hy-CD-O-Pol-4 (**g**,**h**). The isocontour value is 0.02 au (H atom—white sphere, C atom—grey sphere, N atom—blue sphere, O atom—red sphere).

References

1. Lakowicz, J. R. *Principles of Fluorescence Spectroscopy*; Springer US: Boston, MA, 2006. https://doi.org/10.1007/978-0-387-46312-4.
